# A parasite-derived 68-mer peptide ameliorates autoimmune disease in murine models of Type 1 diabetes and multiple sclerosis

**DOI:** 10.1038/srep37789

**Published:** 2016-11-24

**Authors:** Maria E. Lund, Judith Greer, Aakanksha Dixit, Raquel Alvarado, Padraig McCauley-Winter, Joyce To, Akane Tanaka, Andrew T. Hutchinson, Mark W. Robinson, Ann M. Simpson, Bronwyn A. O’Brien, John P. Dalton, Sheila Donnelly

**Affiliations:** 1 The School of Life Sciences, University of Technology Sydney, New South Wales, Australia; 2 The University of Queensland, UQ Centre for Clinical Research, Brisbane, Queensland, Australia; 3 The Centre for Health Technology, University of Technology Sydney, New South Wales, Australia; 4 Medical Biology Center, School of Biological Sciences, Queen’s University, Belfast, Northern Ireland, United Kingdom.

## Abstract

Helminth parasites secrete molecules that potently modulate the immune responses of their hosts and, therefore, have potential for the treatment of immune-mediated human diseases. FhHDM-1, a 68-mer peptide secreted by the helminth parasite *Fasciola hepatica,* ameliorated disease in two different murine models of autoimmunity, type 1 diabetes and relapsing-remitting immune-mediated demyelination. Unexpectedly, FhHDM-1 treatment did not affect the proliferation of auto-antigen specific T cells or their production of cytokines. However, in both conditions, the reduction in clinical symptoms was associated with the absence of immune cell infiltrates in the target organ (islets and the brain tissue). Furthermore, after parenteral administration, the FhHDM-1 peptide interacted with macrophages and reduced their capacity to secrete pro-inflammatory cytokines, such as TNF and IL-6. We propose this inhibition of innate pro-inflammatory immune responses, which are central to the initiation of autoimmunity in both diseases, prevented the trafficking of autoreactive lymphocytes from the periphery to the site of autoimmunity (as opposed to directly modulating their function *per se*), and thus prevented tissue destruction. The ability of FhHDM-1 to modulate macrophage function, combined with its efficacy in disease prevention in multiple models, suggests that FhHDM-1 has considerable potential as a treatment for autoimmune diseases.

The rapid increase in the prevalence of autoimmune disease over the last century cannot be simply attributed to genetic changes, but suggests either the removal of protective factors or the introduction of susceptibility factors in the environment^1^. Several environmental risk factors, such as low vitamin D, infection with Epstein-Barr virus, and smoking, have been associated with the occurrence of autoimmune disease^2^,^3^. However, compelling epidemiological evidence indicates an inverse correlation between infections with helminth parasites and the incidence of autoimmune disease worldwide^4^,^5^,^6^,^7^. These observations concur with the so-called ‘old friends’ hypothesis, suggesting that the long evolutionary co-adaptation between parasites and humans has fundamentally affected the composition of the human immune response. Therefore, by removing the regulatory influences of parasites from human populations attuned to co-exist with them, an imbalance in the immune system is established, thereby increasing susceptibility to immune-mediated disease^8^. Accordingly, helminth parasites likely constitute beneficial commensals rather than harmful pathogens^9^,^10^,^11^.

Indeed, deliberate infection with helminth parasites is being explored as a potential therapy for autoimmune disease. However, the use of live parasites to treat autoimmune disease is not optimal or readily acceptable. Infection undoubtedly causes physiological side effects, due to the parasite’s feeding and migratory activities^12^,^13^. The helminths (*N. americanus* and *T. suis*), shown to be therapeutically beneficial in trials, must be harvested from a mammalian host, a process that inherently carries a risk of contamination with other pathogens. The *T. suis* pig whipworm is not evolved to colonise a human host, and, therefore, is ultimately expelled from the patient. Furthermore, optimal effective therapeutic use of this parasite, demands a regime of 2,500 eggs, delivered orally every two weeks^14^,^15^, which cannot be accurately determined as some eggs fail to produce viable juveniles. There is also a lack of immunological specificity associated with parasitic infection that can suppress immune responses required for immunity to other pathogens and, indeed, vaccines^16^. Thus, a patient receiving helminth therapy is immune-suppressed, just as he/she would be if prescribed general immunosuppressive drugs. A safer and potentially more effective alternative to live infection is to deliver the specific immune-modulatory molecules produced by helminth parasites. This approach would allow the precise mechanisms of action of these molecules to be characterized, and their properties modified to increase therapeutic efficacy. In addition, synthetic molecules can be manufactured to therapeutic standard and can be modified during synthesis to enhance stability and reduce undesirable immunogenicity and toxicity.

Previously, we showed that a two-week treatment of non-obese diabetic (NOD) mice with the medium in which *F. hepatica* adult parasites were maintained (termed excretory-secretory products, FhES) prevented the development of type 1 diabetes (T1D)^17^. Protected animals remained normoglycaemic for up to 30 weeks of age (experimental endpoint; 24 weeks after FhES treatment). Others have also shown that FhES prevented disease development in chronic experimental autoimmune encephalomyelitis (EAE)^18^, a murine model of multiple sclerosis (MS). Fractionation of FhES by gel filtration chromatography identified two abundant secretory molecules, FhHDM-1^19^, a peptide with a cathelicidin-like C-terminal alpha helix, and FhCL1, a cathepsin L cysteine protease^20^,^21^. Here, we tested a synthetic 68-amino acid form of FhHDM-1 and a functionally active recombinant form of FhCL1, for their ability to replicate the protective effect of total FhES and prevent autoimmune disease in mouse models of T1D and MS.

## Results

### FhHDM-1 prevents the development of type 1 diabetes

**Figure 1:**
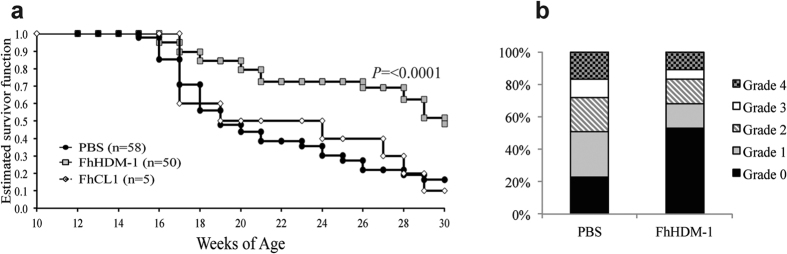
Administration of FhHDM-1 prevents T1D in NOD mice. (**a**) Female NOD/Lt mice were injected i.p. for a total of six times with FhHDM-1, FhCL1 (10 μg in 100 μl sterile PBS), or sterile PBS (100 μl) on alternate days beginning at 4 weeks of age. Mice were then monitored over 30 weeks, assessed for blood glucose weekly, and diagnosed as diabetic after two consecutive measurements >14 mmol/L. Data shown is a combination of four independent experiments. A Tarone-Ware nonparametric test comparing the Kaplan-Meier estimates of survivor function was used to assess significance. (**b**) Pancreas isolated from mice at 13 weeks of age (n = 9) were graded for insulitis on a scale of 0–4; whereby 0 = healthy islet or mild peri-insular mononuclear cell infiltration, 1 = infiltration up to 25% of islet mass, 2 = infiltration up to 50% of islet mass, 3 = infiltration from 50% up to 75% of islet mass, and 4 = less than 25% of islet mass present. The proportion of islets with each grade of insulitis is shown.

Because we have previously shown that FhES can prevent autoimmune disease in mice^17^, the same treatment regime was chosen to assess the efficacy of FhHDM-1 and FhCL1 in preventing T1D in NOD mice. The parasite derived proteins were administered to female NOD mice by intraperitoneal injection beginning at 4 weeks of age (co-incident with the priming of auto-reactive T cell populations and initiation of insulitis), and continued on alternate days for a total of 6 treatments (10 μg/injection). The incidence of diabetes in the vehicle only (PBS treated) cohort was 84%, as expected (Fig. 1a). Administration of FhCL1 afforded no significant protection, with a disease penetrance of 90% observed. By contrast, 50% of mice treated with FhHDM-1 were protected against T1D (*p* < 0.0001 as compared to PBS treated cohorts; Fig. 1a) and remained normoglycaemic at 30 weeks of age (24 weeks after the final injection of FhHDM-1; experimental endpoint). The protection afforded by FhHDM-1 was comparable to that observed using FhES^17^. Examination of H&E stained sections of pancreas isolated from FhHDM-1-treated mice at 13 weeks of age showed a consistent and significant (p < 0.001) reduction in islet inflammation compared to PBS treated mice (Fig. 1b), which is in keeping with the ability of the peptide to prevent autoimmune diabetes.

### FhHDM-1 alleviates the clinical symptoms of relapsing remitting EAE

**Figure 2:**
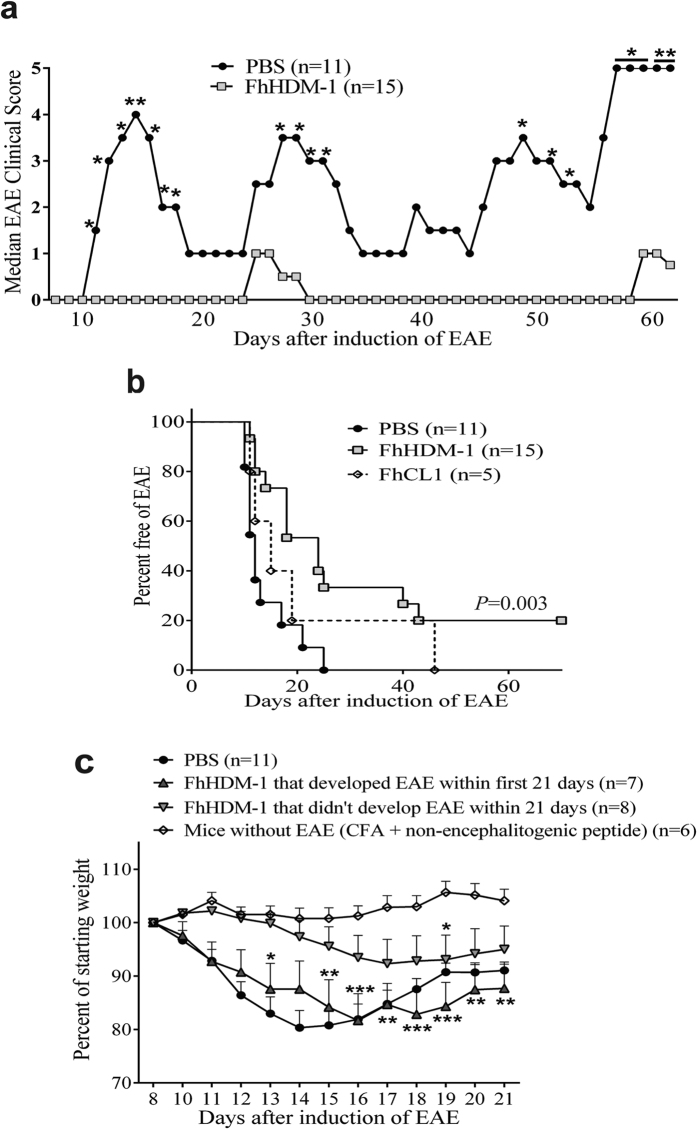
Administration of FhHDM-1 reduces the severity of clinical symptoms in relapsing remitting (RR)-EAE. FhHDM-1, FhCL1 (20 μg in 100 μl sterile PBS), or sterile PBS (100 μl) was injected i.v. into female SJL/J mice every other day for a total of 6 treatments, commencing 3 days prior to the induction of EAE. Following EAE induction, by immunization with 100 μg PLP_139-151_, mice were monitored over 70 weeks, with clinical assessment every 1-2 days using a 5 point scoring scale, with a score of 0 indicating that the animal was free of the clinical signs of EAE, and 5 being moribund. (**a**) The median EAE clinical scores for the PBS treated and FhHDM-1 treated mice are shown. *P < 0.05 **P < 0.01 calculated by non-parametric repeated measure ANOVA. (**b**) Weight loss is shown as a percentage of the starting weight 8 days after induction of EAE, as compared to mice immunized with 100 μg of a non-encephalitogenic peptide (A188) or PLP_139-151_. *P < 0.05 **P < 0.01 ***P < 0.001 calculated by non-parametric repeated measure ANOVA. (**c**) The percentage of mice from (**a**) that remained free of disease over the 70 week period is shown.

To determine whether the prevention of EAE mediated by FhES could also be attributed to FhHDM-1, we tested its efficacy in a relapsing-remitting EAE model in SJL mice. In this model, more than 80% of PBS treated mice had experienced between 2 and 5 attacks by day 70 after EAE induction. By contrast, following intravenous injections of FhHDM-1, mice developed less severe EAE, with fewer relapses per mouse (*p* < 0.0001; Fig. 2a). Seventy days after EAE induction (~60 days after the last injection of FhHDM-1), more than 50% of FhHDM-1 treated mice had experienced only a single attack, and 20% remained completely disease free (Fig. 2b). FhHDM-1 treated mice without EAE exhibited negligible weight loss that was not significantly different to mice treated with the non-encephalitogenic peptide, A188, in Complete Freund’s Adjuvant (CFA). However, mice treated with FhHDM-1, but not protected from EAE, showed weight loss comparable to that seen in PBS treated animals (Fig. 2c). Treatment of mice with FhCL1 was mildly beneficial on the day of EAE onset (Fig. 2b), but it neither significantly reduced the overall disease burden nor the number of relapses.

### FhHDM-1 treatment does not reduce the auto-antigen specific T cell response

**Figure 3:**
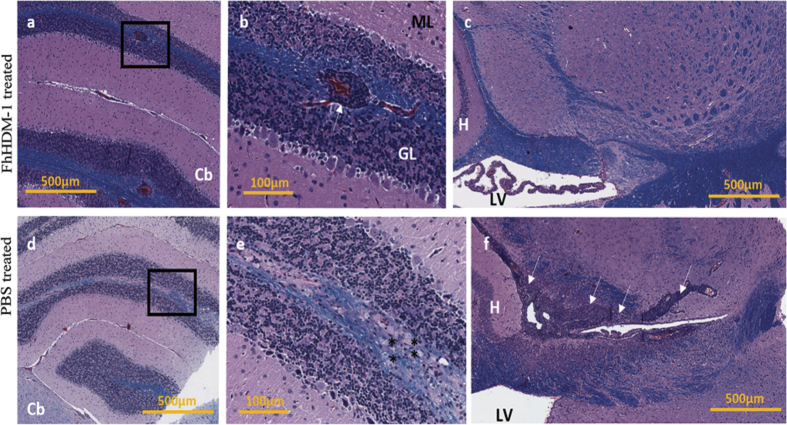
Administration of FhHDM-1 reduces inflammation and demyelination in the CNS in relapsing remitting (RR)-EAE. Histological analysis of brain tissue from (**a**–**c**) FhHDM-1- or (**d**–**f**) PBS-treated EAE mice at 15 day post induction. Paraffin embedded sections were analysed for demyelination by luxol fast-blue/haematoxylin and eosin staining. (**a**) and (**d**) show the cerebellum (Cb), with an enlargement of the area from the black rectangle shown in (**b**,**e**), respectively. The white arrow in (**b**) shows an inflammatory infiltrate that is retained within the perivascular space, with no evidence of surrounding demyelination; this was only seen in the FhHDM-1-treated mice. The asterisks in (**e**) indicate foamy macrophages. (**c**,**f**) show the area near the hippocampus (H) and the lateral ventricle (LV) of the brain, with the white arrows indicating a large area of cellular infiltration and demyelination in (**f**).

**Figure 4:**
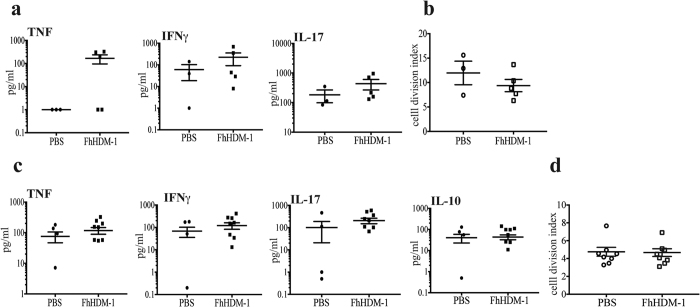
FhHDM-1 treatment does not alter the proliferation or production of cytokines by PLP_139-151_ –specific T cells. (**a**) Lymph nodes were harvested from mice at the first peak of clinical disease (approximately day 15) and (**b**) from mice 70 days after the induction of EAE (shown in Fig. 1b). Single cell suspensions were stained with CFSE and then stimulated with PLP_139-151_ (20 μg/ml) for 72 h. Culture supernatants were collected, and levels of TNF-α, IFN-γ, IL-17, and IL-10 were determined by cytokine bead array assays. Values for each individual mouse were calculated as the average of the experimental triplicate. No IL-10 was detectable in the samples harvested at day 15. (**c**,**d**) Cells were stained with PE-labelled anti-CD4 antibody and the proportion of CD4^+^ CFSE^lo^ (proliferating) cells in the total CD4^+^ CFSE^+^ population was assessed by flow cytometry. Results are shown as the cell division index, which indicates the proportion of proliferating cells in the PLP_139-151_-stimulated cultures/proportion of proliferating cells in the cultures without antigen. Graphs show the means ± SEMs.

Typically, infection with helminth parasites leads to a polarized and potent Thelper (Th)2 type immune response and chronic infection is associated with the differentiation of regulatory T cells (Tregs)^11^,^22^. It is these regulatory responses that are proposed to suppress the development of the detrimental auto-antigen specific Th1 and Th17 responses that mediate autoimmune disease^23^,^24^. Indeed, the protection afforded by FhES in both NOD and EAE mouse models has been attributed to both the secretion of Th2 cytokines and the inhibition of autoantigen specific Th1 type immune responses^17^,^18^. To examine if this was the mechanism of protection mediated by FhHDM-1, draining lymph nodes were harvested from both PBS and FhHDM-1 treated EAE mice when the first peak of disease occurred in the PBS-treated mice (day 15 after disease induction), and PLP_139-151_-specific T cell responses were measured. Despite the observation that FhHDM-1 treated mice displayed less severe clinical symptoms (1.3 ± 0.8) than PBS treated animals (4.1 ± 0.4), and less inflammation and demyelination in the CNS (Fig. 3), there was no apparent difference in the quantity of cytokines or phenotype of immune response produced in response to the auto-antigen (Fig. 4a,b). There was no significant alteration in the levels of the disease mediating pro-inflammatory cytokines, IFNγ, TNF or IL-17 (Fig. 4a), and no increase in the levels of IL-4, IL-5 or IL-10, all of which were negligible in the EAE mice (not shown). Furthermore, analyses of PLP_139-151_-specific responses, among T cells from resident lymph nodes from surviving relapsing remitting (RR)-EAE mice also showed no differences in cytokine production or proliferative responses between PBS and FhHDM-1 treatments (Fig. 4c,d). These results indicated that disease protection by FhHDM-1 was not mediated by the inhibition of auto-antigen specific inflammatory T cell responses or a switch towards a Th2 phenotype. Additionally, FhHDM-1 did not modulate immune responses to injected KLH (Supplementary Fig. 1), thereby verifying that the peptide did not generally suppress antibody production or T helper cell responsiveness.

### FhHDM-1 inhibits the activation of pro-inflammatory macrophages

**Figure 5:**
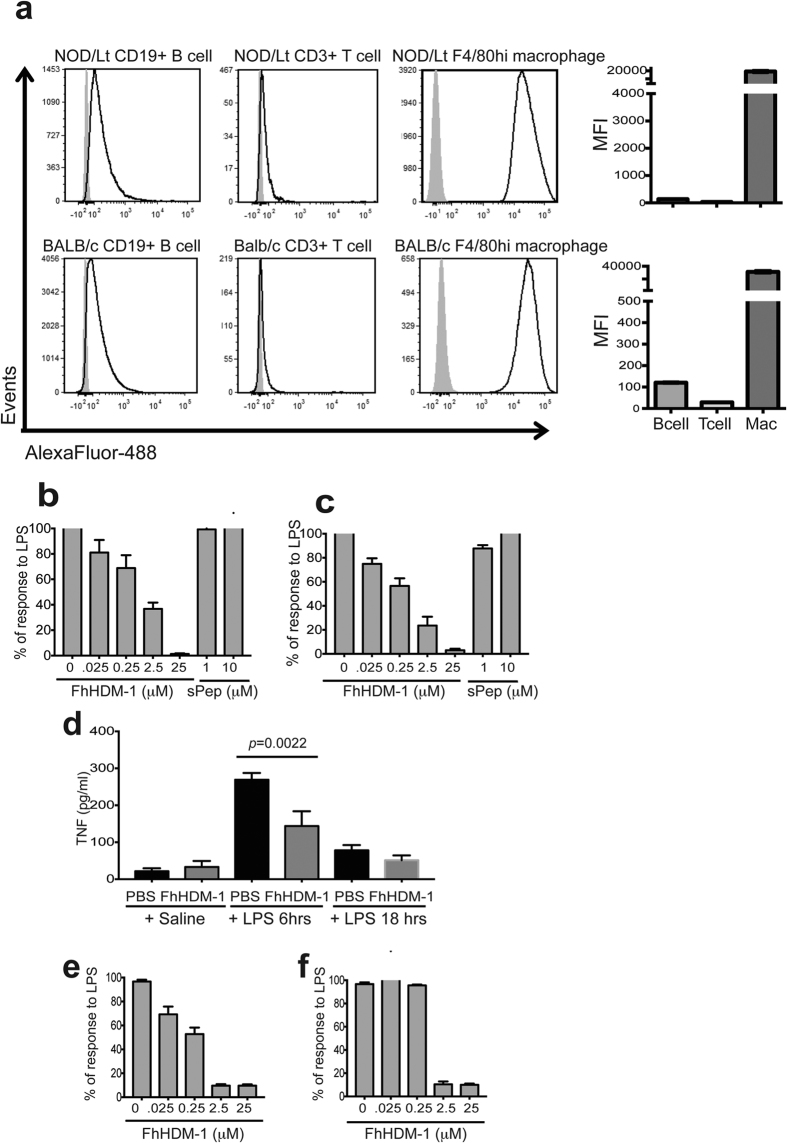
FhHDM-1 interacts with macrophages and inhibits the production of pro-inflammatory cytokines. (**a**) Female NOD/Lt mice (n = 3) or BALB/c mice (n = 3) were injected with fluorescently tagged-FhHDM-1 (10 μg in 100 μl sterile saline) and 20 min later, the peritoneal exudate cells were stained for the identification of T cells, B cells and macrophages by flow cytometry. Panels are representative histograms showing cells isolated from saline treated mice (grey filled) and cells harvested from FhHDM-1 treated mice (black line) within each cell gate and are representative of 4 independent experiments. (**b**,**c**) Macrophages differentiated from the bone marrow of BALB/c mice or (**e**,**f**) from human monocytes were incubated with FhHDM-1 at a range of concentrations (0.025–25 μM) or a scrambled peptide (1 μM or 10 μM) for 1 h and then stimulated by the addition of *E. coli* LPS (10 ng/ml) for 12 h. The quantity of TNF (**b**,**e**) and IL-6 (**c**,**f**) in the supernatant was measured by ELISA. The results are plotted as the means ± SEMs of the % maximal LPS-stimulated TNF released. Each bar represents the mean of 4 independent experiments (total n = 12). (**d**) Peritoneal macrophages were isolated from NOD/Lt mice 24 h after the final injection of FhHDM-1 (n = 10) or saline (n = 9) and stimulated with PBS or *E. coli* LPS (10 ng/ml) for 6 h or 18 h. Cell supernatants were collected and TNF quantified by ELISA. The error bars show the means ± SEMs.

To define the cellular target(s) of FhHDM-1, murine peritoneal exudates were analyzed by flow cytometry 20 mins after the intraperitoneal injection of fluorescently labelled FhHDM-1. This revealed that, of the three most highly represented immune cell populations present in the peritoneal cavity (macrophages, B cells and T cells), FhHDM-1 preferentially bound to macrophages *in vivo* in both NOD mice and immune competent BALB/c mice (Fig. 5a). Since the development of disease in both NOD and EAE mouse models has been shown to require involvement from pro-inflammatory M1-type macrophages we investigated the possibility that FhHDM-1 was exerting its protective effect through the modulation of macrophage activity. The expression levels of co-stimulatory molecules and secretion of cytokines are important determinates of macrophage phenotype and function. While the expression of surface markers (CD40, CD80, CD86, MHC-I, MHC-II) on macrophages was not altered by interaction with FhHDM-1 *in vitro* (Supplementary Fig. 2), the secretion of the pro-inflammatory cytokines, TNF and IL-6, in response to stimulation with bacterial LPS, was inhibited in a dose-dependent manner (Fig. 5b,c). This effect was specifically related to the activity of FhHDM-1, as a scrambled peptide (sPep) exerted no inhibitory effect. This activity was also evident *in vivo* in autoimmune mice. Peritoneal macrophages isolated from FhHDM-1 treated NOD mice secreted significantly reduced levels of TNF in response to LPS *ex vivo* (Fig. 5d). Importantly, in considering the translation of these findings as a treatment for human patients, FhHDM-1 also exerted an inhibitory effect on TNF and IL-6 secretion by human primary macrophages (Fig. 5e,f), an effect that was retained in human whole blood cells (Supplementary Fig. 3). Exposure of macrophages to FhHDM-1, *in vitro* or *in vivo*, did not increase the production of IL-10 or TGFβ, or increase the expression levels of genes specific for an M2 macrophage phenotype (Arg-1, Ym1, Retnlα; data unpublished). Collectively, this data suggests that FhHDM-1 does not alter the phenotype of macrophages, but specifically regulates their pro-inflammatory response.

## Discussion

Studies in animal models and human subjects have demonstrated that infection with helminth parasites can suppress a range of organ specific inflammatory diseases. Infection with either intestinal-dwelling or tissue dwelling helminths produce similar outcomes suggesting a commonality in which they evoke immune-modulatory mechanisms^23^,^24^. We have previously shown that administration of FhES products of the helminth parasite *Fasciola hepatica* prevented the development of T1D in NOD mice^17^. Here, we discovered that a single 68-mer peptide FhHDM-1, which is an abundant component of the FhES, was sufficient to mimic the protective effect of FhES in murine models of T1D and relapsing-remitting EAE. The presence of FhHDM-like molecules in other major human tissue dwelling trematode helminth parasites^19^ suggests that secretion of these peptides may be a common method of immune modulation in these class of helminths, although the mechanism needs to be further investigated.

From the current study, and our previous reports^19^,^25^, we propose that FhHDM-1 modulates the function of macrophages to impair the release of pro-inflammatory cytokines. Although the pathogenesis of autoimmune diseases like T1D and EAE has been demonstrated to be CD4^+^ T cell dependent, innate immune cells, especially macrophages, have important roles in the initiation and progression of disease^26^,^27^. In particular, the differentiation of macrophages towards a pro-inflammatory M1 phenotype underpins the pathophysiology of many autoimmune conditions, including MS, T1D, rheumatoid arthritis, and inflammatory bowel disease^28^,^29^. Inhibition of macrophage function is sufficient to protect mice from auto-reactive T cell driven immune disorders, and elimination of M1 macrophages, or specific inhibition of their pro-inflammatory cytokine production, prevents autoimmune disease in murine models of MS^30^,^31^ and T1D^32^,^33^. As such, macrophages are the central regulators among immune cells determining if autoimmunity is initiated or prevented. The identification of a helminth-derived peptide that regulates macrophage function and results in the modulation of disease progression represents a novel therapeutic approach for diseases involving pro-inflammatory macrophage activity.

While the function of activated macrophages during EAE progression is complicated and not wholly defined, they are known to contribute to the activation of T cells. However, FhHDM-1 did not influence auto-antigen specific T cell responses in RR-EAE mice. Despite this, the presence of primed auto-reactive T cells, although important, may be insufficient to elicit destructive autoimmune responses. Indeed, T cells specific for auto-antigens, such as myelin basic protein or myelin proteolipid protein (autoantigens in MS) and glutamic acid decarboxylase (an autoantigen in T1D) are commonly present in the peripheral blood of healthy individuals^34^,^35^.

Regulation of the pathogenic or tolerogenic activity of T cells is mediated by receptor recognition, in combination with various co-stimulatory and cytokine signals^36^, which are provided by pro-inflammatory macrophages. Adoptive transfer of NOD splenocytes to T-cell deficient recipients had demonstrated that a sustained signal from macrophages is required for pathogenesis^37^; thus, autoreactive T cells transferred to macrophage-depleted recipients had no effect on beta cells suggesting a loss of cytotoxicity but when returned to a macrophage replete environment they regained their ability to destroy beta cells and cause autoimmune diabetes. This re-activation of T cell pathogenicity was shown to require the expression of pro-inflammatory cytokines by macrophages^37^. We suggest, therefore, that the specific modulation of macrophage activity by FhHDM-1 prevents the establishment of conditions required for sustained auto-specific T cell cytotoxicity. However, even if activated, the auto-reactive T cells must traffic from the periphery to the relevant organ to induce disease (pancreas and spinal cord in T1D and EAE, respectively), which normally occurs in response to a pro-inflammatory signal(s). In the case of EAE, depletion of peripheral macrophages, or specifically of TNF production by myeloid cells was insufficient to impact the development of auto-antigen specific Th1 cells, but prevented the infiltration of lymphocytes into the CNS parenchyma, and inhibited demyelination and disease^38^,^39^. In a similar way, peritoneal macrophages were shown to accelerate the disease process in NOD mice^40^ and as a major source of pro-inflammatory cytokines^41^ may also regulate cellular infiltration to the pancreas^42^. Our histological data in both NOD and EAE mice-treated with FhHDM-1, supports a mechanism by which FhHDM-1 regulates the activation of macrophages to consequently reduce the migration of pathogenic immune cells to the site of autoimmunity thereby preventing tissue destruction and subsequent clinical symptoms.

FhHDM-1 and mammalian cathelicidin molecules (murine CRAMP, human LL37) exhibit structural similarity in their C-terminal domains, which is characterized by an amphipathic α-helix region^19^. Accordingly, we have suggested that FhHDM-1 functions as a molecular mimic, whereby it simulates mammalian immune-modulatory mechanisms to interfere with the activity of host innate immune cells^25^. Given this structural similarity, it is plausible that mammalian receptors recognizing cathelicidins also interact with FhHDM-1. It is therefore of interest to note that CRAMP-mediated inhibition of T1D development in mice is dependent upon the expression of epidermal growth factor receptor (EGFR) on pancreatic macrophages^43^. Interestingly, signaling through EGFR has also been shown to enhance oligodendrocyte generation and axonal myelination^44^. The lack of re-myelination observed in patients with relapsing-remitting or secondary progressive forms of MS correlates with lower levels of EGF in the cerebrospinal fluid, as compared to non-clinical controls^45^. Therefore, interaction with EGFR may be the common mechanism by which FhHDM-1 prevents the development of distinct autoimmune diseases.

Importantly, FhHDM-1 exhibits significantly less cytotoxicity^46^ compared to endogenously produced mammalian cathelicidins, suggesting a safer toxicity profile and greater potential as a therapeutic. Furthermore, the translation of LL37 to a human immune therapeutic is hampered by its susceptibility to degradation by proteases^47^,^48^. In contrast, FhHDM-1 is resistant to proteolytic cleavage by human proteases^19^,^25^, suggesting increased stability and a more attractive pharmacokinetic profile. Furthermore, the activity of FhHDM-1 is solely anti-inflammatory, as opposed to the mixture of anti- and pro-inflammatory activities exhibited by mammalian cathelicidins^49^. This characteristic is consistent with its putative function in *F. hepatica* infection, whereby FhHDM-1 dampens pro-inflammatory immune responses in the host to ensure parasite longevity. The ability of the FhHDM-1 peptide alone to protect the integrity of host tissue and prevent the development of the dysregulated pro-inflammatory responses that initiate autoimmunity identifies it as a novel parasite-derived therapeutic for human autoimmune/inflammatory diseases. Importantly, only a two-week systemic treatment of FhHDM-1 was sufficient to provide long-term disease prevention (up to 30 weeks, experimental endpoint) in both models of autoimmune disease when delivered at, or prior to, the onset of disease. While this efficacy represents an improved treatment regime to many current therapies of autoimmune conditions, a demonstration of effectiveness in established disease would make FhHDM-1 a very attractive candidate as a therapeutic. Considering that administration of FhES, of which FhHDM-1 is an abundant component, prevents the progression of chronic EAE when delivered at the onset of symptoms^18^, it is very likely that FhHDM-1 will prove to be an effective therapy to halt the progression of established autoimmune disease. This idea is currently being investigated.

This study provides proof-of-concept in two pre-clinical rodent models that a single parasite-derived peptide offers a novel treatment for individuals predisposed to, or suffering from, autoimmune disease. This finding adds to the growing body of evidence that molecules secreted by helminth parasites offer a unique resource for mining of anti-inflammatory drugs^50^,^51^,^52^. The efficacy of parasite-derived molecules has been fine-tuned over millennia of co-evolution with humans, suggesting that the pharmacological activity of these peptides has already been optimized over time by nature. Therefore, exploiting these helminth proteins offers the potential for drug treatments that are far superior to current biologicals whose widespread use is limited by a lack of specificity and a range of adverse side effects.

## Material and Methods

### Parasite-derived proteins

Functionally active recombinant *F. hepatica* cathepsin L1 (FhCL1) was expressed in *Pichia pastoris* and purified by affinity chromatography on nickel-nitrilotriacetic acid-agarose as previously described^21^. FhHDM-1 and a scrambled peptide (sequences shown in Supplementary Fig. 4) were chemically synthesized endotoxin-free by GLBiochem (Shanghai, China)^19^.

FhHDM-1 was fluorescently labelled using Alexa Fluor 488 NHS Ester (Thermofisher Scientific), according to the manufacturer’s instructions. Briefly, FhHDM-1 was incubated with a 10-fold molar excess of dye overnight, and then dialyzed against sterile endotoxin-free saline (Baxter Healthcare) to remove excess unconjugated dye.

### NOD mouse model

FhHDM-1 or FhCL1 (10 μg in 100 μl sterile PBS) was administered intraperitoneally (i.p) to four-week old female NOD/Lt mice (Animal Resources Centre, WA, Australia) on alternate days for a total of 6 injections. Control mice received 100 μl of sterile PBS. Glucose levels were measured from tail vein blood weekly, from 13 weeks of age, using Accu-check Advantage blood glucose strips (Roche). Animals were sacrificed at diabetes onset (defined by two consecutive blood glucose concentrations above 14 mmol/L). To quantify insulitis, formalin-fixed paraffin-embedded pancreata were sectioned (4 μm) at three non-overlapping levels, such that each section was separated from the preceding one by at least 20 μm. Sections were stained by hematoxylin and eosin (H&E), studied for their histological characteristics, and graded for insulitis on a scale of 0–4; whereby 0 = healthy islet or mild peri-insular mononuclear cell infiltration, 1 = infiltration up to 25% of islet mass, 2 = infiltration up to 50% of islet mass, 3 = infiltration from 50% up to 75% of islet mass, and 4 = less than 25% of islet mass present. Slides were assessed in a blinded fashion and all islets in 10 slides from each pancreas were scored. Ethical approval for this study was granted by the University of Technology Sydney (UTS) Animal Care and Ethics Committee (Approval Number: 2010-432A) and experiments were conducted in accordance with the approved guidelines.

### EAE induction and clinical and histological assessment

To induce EAE, 50 μg of PLP_139-151_ in an emulsion consisting of equal volumes of PBS and CFA containing an additional 4 mg/ml of *Mycobacterium tuberculosis* H37Ra (Difco) was injected subcutaneously (s.c.) into six to eight-week old SJL/J mice (Animal Resources Centre, WA, Australia). Each mouse also received 300 ng of *Bordetella pertussis* toxin (Sapphire Biosciences) intravenously (i.v.) on days 0 and 3. FhHDM-1, FhCL1 (10 μg in 100 μl sterile PBS) or sterile PBS (100 μl) was injected i.v. into female SJL/J mice every other day for a total of 6 treatments, commencing 3 days prior to the induction of EAE. Following EAE induction, mice were monitored over 70 days. Clinical assessment (weight and observation) was conducted daily from day 7 post-injection. Mice were scored according to the following criteria: 0, no disease; 1, decreased tail tone or slightly clumsy gait; 2, tail atony and/or moderately clumsy gait and/or poor righting ability; 3, hind limb weakness; 4, hind limb paralysis; and 5, moribund state. For histological assessment of CNS tissue, brains and spinal cords were removed from mice at the time of the peak of the first attack of disease in control mice, fixed in 10% formalin, embedded in paraffin and 8 μm sections were cut and stained with Luxol fast blue/H&E. Ethical approval for this study was granted by the University of Queensland Animal Ethics Committee (Approval Number: 2010-432 A) and experiments conducted in accordance with the approved guidelines.

### T cell proliferation assays

Dispersed lymph node cells harvested from individual mice were washed twice with sterile PBS and stained with 2 μmol/l CFSE (Invitrogen Life Technologies) for 30 min at 37 °C. Cells were cultured in the presence or absence of PLP_139-151_peptide (20 μg/ml) for 3 d. Wells containing no peptide and Concanavalin A (2 mg/ml) were used as negative and positive controls, respectively. After 3 d, cells were harvested and supernatants were collected for cytokine bead arrays (CBA). Cells were washed with PBS containing 1% FCS and 0.01% sodium azide (wash buffer) (Sigma-Aldrich), before staining with PerCP-labeled anti-CD4 antibody (BD Biosciences, Australia) for 1 h at 4 °C. PerCP-labeled isotype-matched primary antibody (BD Biosciences) was used as a negative control. Stained cells were analyzed on a FACSCalibur flow cytometer using CellQuest Software (BD Biosciences). All staining profiles were based on lymphocyte-gated cells, as determined by forward and side scatter properties. The percentage of proliferating CD4^+^ T cells in the sample (CFSE^lo^) was assessed, and a cell division index was calculated by dividing the percentage of dividing cells in the PLP-stimulated group by the percentage of cells dividing in the control group (no peptide).

Levels of cytokines in culture supernatants from control or PLP-stimulated splenocytes were quantified using CBA (mouse TNF, bead C8; IL-10, bead C4; IL-17, bead C5; IFN-γ, bead A4), as per the manufacturer’s instructions (BD Biosciences).

### Anti-KLH antibody response

BALB/c mice were given 6 i.p. injections of FhHDM-1 (10 μg), delivered on alternate days. Mice were then challenged with a single i.p. delivery of KLH (100 μg; without adjuvant; Sigma) midway through or 10 days after the FhHDM-1 treatment regime. Sera were collected by cardiac puncture at day 7 (for anti-KLH IgM) or day 20 (for anti-KLH IgG) and the levels of KLH-specific antibodies were quantified by end-point titration and comparison to a pooled sample of sera from untreated mice. The end-point was defined as the highest dilution of sera that gave an optical density of 4 standard deviations above the negative/untreated control.

### Cell binding studies

Four-week old female NOD/Lt mice and six-week old BALB/c mice were given a single i.p. injection of 10 μg (in 100 μl sterile saline) of Alexa Fluor-488 FhHDM-1, or vehicle control. After 20 min, peritoneal lavages were collected in 5 ml ice-cold FACS staining wash (PBS/1%BSA/2%HI FCS/0.05% NaN_3_). Peritoneal exudate cells were first blocked with Mouse BD Fc Block as per the manufacturer’s instructions (BD), and then stained for flow cytometry with combinations of anti-mouse CD3 (BD; clone 17A2), anti-mouse CD19 (BD; clone 1D3) and anti-mouse F4/80 (Invitrogen; clone BM8), to identify T cells, B cells and macrophages, respectively, at concentrations recommended by the manufacturer. Data was acquired using the BD Fortessa X20 (BD Biosciences) using FACS Diva software (BD Biosciences). Analysis was performed using FCS Express 4 Flow Research Edition (*De Novo* Software).

### Expression of surface markers of macrophage activation

Primary (CD11b^+^F4/80^+^) murine macrophages were derived from the bone marrow of BALB/c mice, by culturing with M-CSF (50ng/ml; eBioscience) for 6 days, and then seeded in RPMI supplemented with 10%v/v FCS and 50 μM beta mercaptoethanol at a density of 1 × 10^6^ cells/ml. Macrophages were untreated or incubated with FhHDM-1 (50 μg/mL) or LPS (100 ng/ml) overnight. For detection of MHCII expression only, macrophages were left untreated or incubated with FhHDM-1 (50 μg/ml) or IFNγ (10 ng/ml) for 2 h. Cells were blocked with Mouse Fc Block (BD Biosciences), and then stained with antibodies: anti-mouse CD80 (BD; clone 16-10A1), anti-mouse CD86 (BD; clone GL1), anti-mouse CD40 (BD; clone 3/23), anti-mouse I-A/I-E (BD; clone M5/114.15.2) or anti-mouse H-2K[d] (BD; clone SF1-1.1) to identify expression of CD80, CD86, CD40, MHCII and MHCI, respectively. Samples stained with corresponding isotype controls were included. Data was acquired using the BD LSR II (BD Biosciences) using FACS Diva software (BD Biosciences). Analysis was performed using FCS Express 4 Flow Research Edition (*De Novo* Software).

### TNF and IL-6 secreted by murine and human macrophages

Primary murine macrophages were derived from bone marrow as described above. Primary human macrophages were derived from monocytes isolated from buffy coats obtained from normal healthy adult donors and supplied by the Australian Red Cross Blood Service. Briefly, mononuclear cells were purified from buffy coats by Ficoll-Paque separation, and CD14^+^ monocytes enriched by positive selection (Miltenyi Biotec). CD14^+^ monocytes were differentiated into macrophages over 7 days in Iscove’s Modified Dulbecco’s Medium (IMDM), supplemented with 2% v/v human serum and differentiation was confirmed by flow cytometry.

For assays in which whole blood was used, 10 ml samples of blood, isolated from normal healthy donors (Australian Red Cross), were diluted to 50 ml in RPMI, and washed twice (400 × g, 5 min). A further two washes in RPMI media (130 × g, 5 mins) were performed to remove platelets. The resulting cell preparation was resuspended in RPMI and plated at a concentration of 1 × 10^6^ cells/ml.

Cell preparations (1 × 10^6^ cells/ml) were incubated with a range of concentrations of FhHDM-1 peptides for 1 h at 37 °C, and then stimulated with *E. coli* LPS (10 ng/ml; from *E. coli* 0111.B4; Sigma Aldrich) overnight at 37^o^C/5% CO_2_. The amount of TNF and IL-6 in supernatants was measured by ELISA (BD Biosciences).

### TNF secretion from peritoneal macrophages *ex vivo*

Peritoneal lavages were collected from female NOD/Lt mice treated with FhHDM-1, or vehicle control, 24 h after the final dose. Macrophages were isolated from peritoneal exudate cells (PECs) by adherence to plastic over 60 min in serum free media. Following a rest period of 30 min, cells were stimulated with LPS (100 ng/ml, from *E. coli* 0111.B4; Sigma Aldrich) or vehicle control, overnight in culture. TNF in the culture supernatant was measured by ELISA using the mouse TNF ELISA Set II (BD Biosciences).

### Statistical Analysis

P values were calculated using the Student’s t test for normally distributed data. The differences in clinical symptoms between treatment groups was analyzed using either a non-parametric Tarone Ware test or a non-parametric repeated measure ANOVA as indicated in the text.

## Additional Information

**How to cite this article**: Lund, M. E. *et al*. A parasite-derived 68-mer peptide ameliorates autoimmune disease in murine models of Type 1 diabetes and multiple sclerosis. *Sci. Rep.* **6**, 37789; doi: 10.1038/srep37789 (2016).

**Publisher's note:** Springer Nature remains neutral with regard to jurisdictional claims in published maps and institutional affiliations.

## Supplementary Material

Supplementary Figures
