# Supplementary material for: A parasite-derived 68-mer peptide ameliorates autoimmune disease in murine models of Type 1 diabetes and multiple sclerosis
Source: Sci Rep. 2016 Nov 24;6:37789. doi: 10.1038/srep37789 (PMC5121616; doi:10.1038/srep37789)
Supplement: Supplementary Figures [file srep37789-s1.docx]

**Supplementary Information**

**A parasite-derived 68-mer peptide ameliorates autoimmune disease in murine models of Type 1 diabetes and multiple sclerosis.**

Maria E. Lund^1†^, Judith Greer^2†^, Aakanksha Dixit^2^, Raquel Alvarado^1^, Padraig McCauley-Winter^1^, Joyce To^1^, Akane Tanaka^1^, Andrew T. Hutchinson^1,3^, Mark W. Robinson^4^, Ann M. Simpson^1,3^, Bronwyn A. O’Brien^1,3^, John P. Dalton^4^, Sheila Donnelly^1*^

^1^The School of Life Sciences, University of Technology Sydney, New South Wales, Australia; ^2^The University of Queensland, UQ Centre for Clinical Research, Brisbane, Queensland, Australia; ^3^The Centre for Health Technology, University of Technology Sydney, New South Wales, Australia; ^4^Medical Biology Center, School of Biological Sciences, Queen's University, Belfast, Northern Ireland.

† These authors contributed equally

*Correspondence should be addressed to S.D. (email: Sheila.Donnelly@uts.edu.au)

**Supplementary Figure 1.**

**FhHDM-1 treatment does not alter the production of KLH specific antibodies**





Female BALB/c mice (n=10) were given 6 i.p. injections of FhHDM (10 µg in 100 µl sterile PBS) delivered on alternate days and challenged with a single i.p injection of KLH (100 μg; without adjuvant) either after the third FhHDM-1 injection (KLH co-inj) or 10 days after the final FhHDM-1 injection (KLH post-inj). The levels of anti-KLH IgM and IgG were quantified by end-point titration in comparison to a pooled sample of sera from untreated mice and displayed as means ± SEMs.

**Supplementary Figure 2.**

**
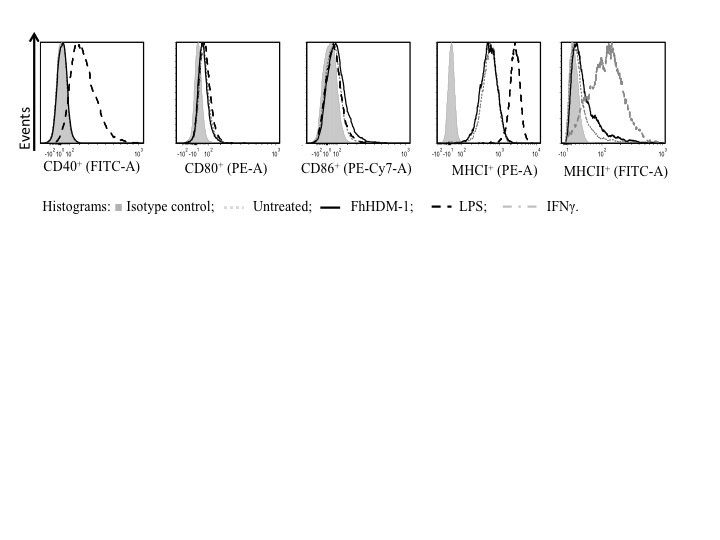
Treatment of macrophages with FhHDM-1 does not alter the expression levels of activation surface markers**

BALB/c BMDMs were left untreated or incubated with FhHDM-1 (50 µg/ml) or LPS (as a positive control; 100 ng/ml) overnight. The expression levels of surface markers of activation were assessed by flow cytometry using specific antibodies and corresponding isotype controls. For detection of MHCII expression only, BMDMs were left untreated or incubated with FhHDM-1 (50 µg/ml) or IFNγ (positive control; 10 ng/ml) for 2h. Histograms are representative of two individual experiments performed in duplicate for each sample treatment.

**Supplementary Figure 3.**

**FhHDM-1 reduces the production of IL-6 from human whole blood in response to stimulation with LPS.**





Samples of whole blood from healthy human donors (n=5; in triplicate for each sample) were treated with serial dilutions (log10 dilutions from 25 µM) of FhHDM-1 for 1h at 37^o^C, and then stimulated with *E. coli* LPS (10 ng/ml). The amount of IL-6 secreted by the cells was quantified by ELISA.

**Figure 4: Amino acid sequence of the full length FhHDM-1 and the scrambled peptide (sPep)**

**FhHDM-1**

SEESREKLRESGRKMVKALRDAVTKAYEKARDRAMAYLAKDNLGEKITEVITILLNRLTDRLEKYAGN

**sPep**

LGFRSEIKFRVRKFRLPTSLDFKKKGEKIQIDLN

The functional c-terminal amphipathic helix (underlined) of FhHDM-1 is homologous with the mammalian cathelicidin, LL-37. A scrambled version of the LL37 peptide (sPep), which has previously been shown to be inactive in cellular assays, was used as a control peptide.
